# 4-[5-(4-Benzoyl­oxyphen­yl)-1,2,4-oxa­diazol-3-yl]phenyl benzoate

**DOI:** 10.1107/S1600536813006922

**Published:** 2013-03-16

**Authors:** B. C. Manjunath, S. Madan Kumar, K. S. Vinay Kumar, M. Prabhuswamy, M. P. Sadashiva, N. K. Lokanath

**Affiliations:** a Department of Studies in Physics, Manasagangotri, University of Mysore, Mysore, 570 006, India; b Department of Studies in Chemistry, Manasagangotri, University of Mysore, Mysore, 570 006, India

## Abstract

In the title compound, C_28_H_18_N_2_O_5_, the dihedral angle between the terminal benzoate rings is 20.67 (12)°. The central oxadiazole ring is almost coplanar with its two benzene ring substituents, making dihedral angles of 4.80 (16) and 5.82 (16)°. In the crystal, pairs of C—H⋯O hydrogen bonds form inversion dimers with *R*_2_^2^(40) ring motifs. The structure also features C—H⋯O, C—H⋯π and π–π inter­actions [centroid–centroid separation = 3.695 (4) Å].

## Related literature

For the use of oxadiazole derivatives as anti­microbial agents, see: Dhol *et al.* (2005 ▶) and for a related structure, see: Emmerling *et al.* (2006 ▶). For hydrogen-bond motifs, see: Bernstein *et al.* (1995 ▶).
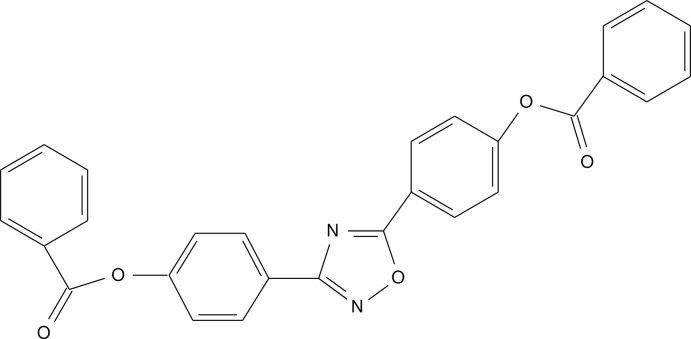


## Experimental

### 

#### Crystal data

C_28_H_18_N_2_O_5_
*M* *_r_* = 462.44
Monoclinic, 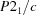

*a* = 21.069 (18) Å
*b* = 6.063 (5) Å
*c* = 18.727 (16) Å
β = 107.159 (13)°
*V* = 2286 (3) Å^3^
*Z* = 4
Mo *K*α radiation
μ = 0.09 mm^−1^
*T* = 300 K
0.23 × 0.23 × 0.22 mm

#### Data collection

Oxford Diffraction Xcalibur Eos diffractometer
21331 measured reflections
4513 independent reflections
2518 reflections with *I* > 2σ(*I*)
*R*_int_ = 0.060

#### Refinement

*R*[*F* ^2^ > 2σ(*F* ^2^)] = 0.068
*wR*(*F* ^2^) = 0.191
*S* = 1.03
4513 reflections
316 parameters
H-atom parameters constrained
Δρ_max_ = 0.38 e Å^−3^
Δρ_min_ = −0.22 e Å^−3^

### 

Data collection: *CrysAlis PRO* (Oxford Diffraction, 2009 ▶); cell refinement: *CrysAlis PRO*; data reduction: *CrysAlis PRO*; program(s) used to solve structure: *SHELXS97* (Sheldrick, 2008 ▶); program(s) used to refine structure: *SHELXL97* (Sheldrick, 2008 ▶); molecular graphics: *Mercury* (Macrae *et al.*, 2008 ▶); software used to prepare material for publication: *SHELXL97* and *Mercury*.

## Supplementary Material

Crystal structure: contains datablock(s) global, I. DOI: 10.1107/S1600536813006922/sj5303sup1.cif

Structure factors: contains datablock(s) I. DOI: 10.1107/S1600536813006922/sj5303Isup2.hkl

Supplementary material file. DOI: 10.1107/S1600536813006922/sj5303Isup3.cml

Additional supplementary materials:  crystallographic information; 3D view; checkCIF report

## Figures and Tables

**Table 1:** Hydrogen-bond geometry (Å, °) *Cg*3 is the centroid of the C10–C15 ring.

*D*—H⋯*A*	*D*—H	H⋯*A*	*D*⋯*A*	*D*—H⋯*A*
C18—H18⋯O5^i^	0.93	2.57	3.407 (5)	150
C25—H25⋯O3^ii^	0.93	2.34	3.222 (6)	158
C28—H28⋯*Cg*3^iii^	0.93	2.96	3.678 (5)	135

Symmetry codes: (i) 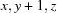; (ii) 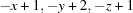; (iii) 
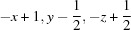
.

**Fig. 1.**
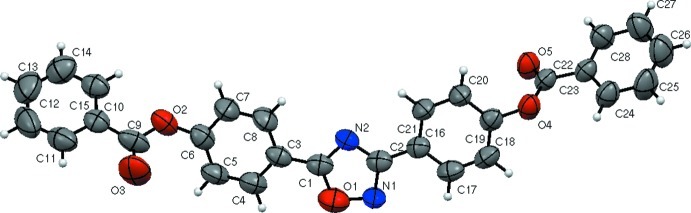
ORTEP diagram of the title molecule with 50% probability ellipsoids.

**Fig. 2.**
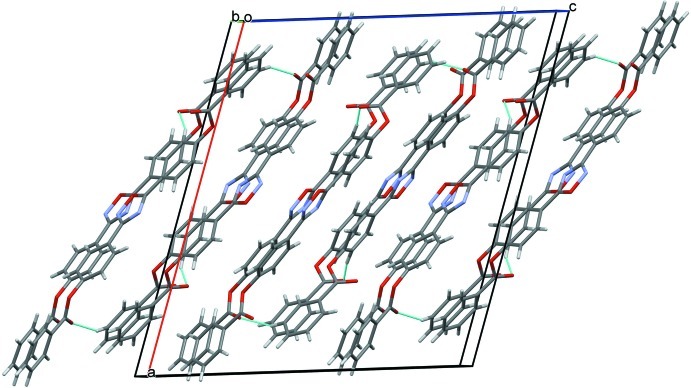
Packing diagram of molecule, viewed along the crystallographic b axis. Dotted lines represent intermolecular hydrogen bonds.

## supplementary crystallographic information

### Comment

Oxadiazole derivatives are known to act as antimicrobial agents (Dhol, *et al.*, 2005). In the title oxadiazole derivative, C_28_H_18_N_2_O_5_, (Fig. 1), the geometry of the oxodiazole ring is comparable to that found in similar molecules (Emmerling *et al.*, 2006). The dihedral angle between the two terminal benzoate rings is 20.67 (12)°. The central oxadiazole ring and its two benzene ring substituents are almost co-planar with the dihedral angles between the O1/C1/N1/C2/N2/ oxadiazole ring and the C3/C4/C5/C6/C7/C8 and C16/C17/C18/C19/C20/C21 benzene rings are 4.80 (16)° and 5.82 (16)° respectively.

C25–H25···O3 hydrogen bonds (Table 1) link adjacent molecules to form inversion dimers with *R*^2^_2_(40) ring motifs (Bernstein *et al.*, 1995). An additional C18–H18···O5 contact together with a C28—H28···*Cg*3 interaction (Table 1) and a Cg1···Cg2^iv^*π*···*π* contact with a centroid-centroid distance of 3.695 (4) Å further stabilise the packing. Cg1 and Cg2 are the centroids of the O1/N1/C1/C2/N2 and C3/C4/C5/C6/C7/C8 rings, respectively and ^iv^ = -*x* + 1, -*y* - 1/2, -*z* + 1.

### Experimental

4-[(*E*)-(hydroxyimino)methyl] phenyl benzoate (4.8 mmole) was dissolved in chloroform, *N*-chlorosuccinimide (5.2 mmole) was added followed by slow addition of sodium carbonate (8.8 mmole) at room temperature. Then, the resulting reaction mixture was stirred for up to 18 h. After completion of reaction (monitored by TLC), the reaction mixture was diluted with water (50 ml). The aqueous layer was extracted with ethyl acetate (3*20 ml), the combined ethyl acetate layer was washed with brine solution (2*25 ml). Then, the organic layer was dried over anhydrous sodium sulfate, filtered and concentrated under reduced pressure to afford the crude product, which was further purified by column chromatography over silica gel (60–120 mesh) using hexane:ethyl acetate mixture in an 8:2 ratio as eluent. The pure compound was crystallized from ethyl acetate and hexane, to give white single crystals.

^1^H NMR (DMSO-d6, 300 MHz): δ 7.33 (t, J=7.2 Hz, 2H), 7.13 (T, J=8.7 Hz, 4H), 7.02 (t, J=7.2 Hz, 2H), 6.75 (s, 2H), 4.51 (d, J=1.8 Hz, 2H), 3.08 (s, 1H).

Mass: Calc. 231.3 found: 232 (*M*+1). Melting point (°C): 90 (Uncorrected)

### Refinement

All hydrogen atoms were located geometrically with C—H = 0.93–0.97) Å and allowed to ride on their parent atoms with *U*_iso_(H) = 1.2*U*_eq_(aromatic C) or 1.5*U*_iso_(methyl C).

### Crystal data

**Table:**

C_28_H_18_N_2_O_5_	*F*(000) = 960
*M**_r_* = 462.44	*D*_x_ = 1.344 Mg m^−^^3^
Monoclinic, *P*2_1_/*c*	Mo *K*α radiation, λ = 0.71073 Å
Hall symbol: -P 2ybc	Cell parameters from 4513 reflections
*a* = 21.069 (18) Å	θ = 2.0–26.0°
*b* = 6.063 (5) Å	µ = 0.09 mm^−^^1^
*c* = 18.727 (16) Å	*T* = 300 K
β = 107.159 (13)°	Needle, white
*V* = 2286 (3) Å^3^	0.23 × 0.23 × 0.22 mm
*Z* = 4	

### Data collection

**Table:**

Oxford Diffraction Xcalibur Eos diffractometer	2518 reflections with *I* > 2σ(*I*)
Radiation source: fine-focus sealed tube	*R*_int_ = 0.060
Graphite monochromator	θ_max_ = 26.0°, θ_min_ = 2.0°
Detector resolution: 16.0839 pixels mm^-1^	*h* = −26→26
ω scans	*k* = −7→7
21331 measured reflections	*l* = −23→23
4513 independent reflections	

### Refinement

**Table:**

Refinement on *F*^2^	Primary atom site location: structure-invariant direct methods
Least-squares matrix: full	Secondary atom site location: difference Fourier map
*R*[*F*^2^ > 2σ(*F*^2^)] = 0.068	Hydrogen site location: inferred from neighbouring sites
*wR*(*F*^2^) = 0.191	H-atom parameters constrained
*S* = 1.03	*w* = 1/[σ^2^(*F*_o_^2^) + (0.0786*P*)^2^ + 0.5948*P*] where *P* = (*F*_o_^2^ + 2*F*_c_^2^)/3
4513 reflections	(Δ/σ)_max_ < 0.001
316 parameters	Δρ_max_ = 0.38 e Å^−^^3^
0 restraints	Δρ_min_ = −0.22 e Å^−^^3^

### Special details

**Table:**

Geometry. Bond distances, angles *etc*. have been calculated using the rounded fractional coordinates. All su's are estimated from the variances of the (full) variance-covariance matrix. The cell e.s.d.'s are taken into account in the estimation of distances, angles and torsion angles
Refinement. Refinement on *F*^2^ for ALL reflections except those flagged by the user for potential systematic errors. Weighted *R*-factors *wR* and all goodnesses of fit *S* are based on *F*^2^, conventional *R*-factors *R* are based on *F*, with *F* set to zero for negative *F*^2^. The observed criterion of *F*^2^ > σ(*F*^2^) is used only for calculating -*R*-factor-obs *etc*. and is not relevant to the choice of reflections for refinement. *R*-factors based on *F*^2^ are statistically about twice as large as those based on *F*, and *R*-factors based on ALL data will be even larger.

### Fractional atomic coordinates and isotropic or equivalent isotropic displacement parameters (Å^2^)

**Table:**

	*x*	*y*	*z*	*U*_iso_*/*U*_eq_	
O1	0.53828 (13)	1.1931 (4)	0.31832 (14)	0.1027 (11)	
O2	0.76931 (12)	0.6953 (4)	0.21732 (12)	0.0864 (9)	
O3	0.84189 (14)	0.9715 (5)	0.25694 (17)	0.1262 (12)	
O4	0.29559 (10)	0.7471 (3)	0.52085 (12)	0.0762 (8)	
O5	0.25276 (10)	0.4910 (4)	0.43300 (12)	0.0811 (8)	
N1	0.48512 (12)	1.1945 (4)	0.35022 (14)	0.0723 (10)	
N2	0.53434 (12)	0.8645 (4)	0.36922 (13)	0.0652 (9)	
C1	0.56466 (16)	0.9925 (5)	0.33120 (16)	0.0686 (11)	
C2	0.48632 (15)	0.9947 (5)	0.37851 (16)	0.0662 (11)	
C3	0.62017 (15)	0.9293 (5)	0.30344 (16)	0.0671 (11)	
C4	0.64460 (17)	1.0655 (5)	0.25813 (18)	0.0801 (12)	
C5	0.69506 (19)	0.9989 (6)	0.23065 (18)	0.0837 (14)	
C6	0.72168 (16)	0.7925 (6)	0.24910 (17)	0.0753 (11)	
C7	0.69841 (17)	0.6508 (6)	0.29357 (19)	0.0867 (14)	
C8	0.64812 (17)	0.7210 (6)	0.32118 (19)	0.0828 (12)	
C9	0.8266 (2)	0.7982 (6)	0.22570 (19)	0.0887 (14)	
C10	0.87083 (16)	0.6675 (6)	0.19119 (17)	0.0716 (11)	
C11	0.9304 (2)	0.7655 (7)	0.1915 (2)	0.1027 (17)	
C12	0.9727 (2)	0.6540 (10)	0.1601 (3)	0.135 (3)	
C13	0.9549 (3)	0.4485 (10)	0.1288 (3)	0.133 (3)	
C14	0.8977 (2)	0.3540 (7)	0.1302 (2)	0.1070 (17)	
C15	0.85597 (18)	0.4614 (6)	0.16063 (19)	0.0853 (12)	
C16	0.43672 (14)	0.9309 (5)	0.41587 (16)	0.0626 (10)	
C17	0.38540 (16)	1.0725 (5)	0.41790 (18)	0.0765 (11)	
C18	0.33900 (16)	1.0085 (5)	0.45203 (18)	0.0768 (12)	
C19	0.34337 (14)	0.8044 (5)	0.48421 (17)	0.0669 (11)	
C20	0.39386 (14)	0.6606 (5)	0.48425 (17)	0.0707 (11)	
C21	0.44033 (14)	0.7249 (5)	0.44904 (18)	0.0712 (11)	
C22	0.25088 (13)	0.5876 (5)	0.48832 (17)	0.0610 (10)	
C23	0.20080 (14)	0.5533 (5)	0.52860 (15)	0.0637 (10)	
C24	0.18931 (16)	0.7044 (6)	0.57868 (19)	0.0821 (12)	
C25	0.14077 (19)	0.6633 (7)	0.6137 (2)	0.1033 (17)	
C26	0.1046 (2)	0.4723 (8)	0.5987 (2)	0.1142 (19)	
C27	0.1155 (2)	0.3225 (7)	0.5490 (2)	0.1099 (17)	
C28	0.16333 (17)	0.3616 (6)	0.51374 (19)	0.0847 (12)	
H4	0.62630	1.20520	0.24610	0.0960*	
H5	0.71090	1.09150	0.20020	0.1010*	
H7	0.71640	0.51040	0.30460	0.1040*	
H8	0.63270	0.62840	0.35200	0.0990*	
H11	0.94170	0.90420	0.21260	0.1230*	
H12	1.01290	0.71690	0.15990	0.1620*	
H13	0.98290	0.37480	0.10660	0.1590*	
H14	0.88690	0.21390	0.11010	0.1280*	
H15	0.81630	0.39450	0.16090	0.1020*	
H17	0.38250	1.21130	0.39600	0.0920*	
H18	0.30470	1.10360	0.45330	0.0920*	
H20	0.39680	0.52360	0.50730	0.0850*	
H21	0.47430	0.62860	0.44770	0.0860*	
H24	0.21410	0.83370	0.58900	0.0980*	
H25	0.13290	0.76530	0.64710	0.1240*	
H26	0.07250	0.44430	0.62250	0.1370*	
H27	0.09040	0.19370	0.53890	0.1320*	
H28	0.17050	0.25920	0.48000	0.1010*	

### Atomic displacement parameters (Å^2^)

**Table:**

	*U*^11^	*U*^22^	*U*^33^	*U*^12^	*U*^13^	*U*^23^
O1	0.124 (2)	0.0824 (18)	0.0989 (18)	−0.0007 (15)	0.0286 (16)	0.0218 (14)
O2	0.0992 (16)	0.0847 (16)	0.0788 (15)	−0.0287 (13)	0.0317 (13)	−0.0061 (12)
O3	0.134 (2)	0.108 (2)	0.140 (2)	−0.0487 (18)	0.0455 (19)	−0.0497 (19)
O4	0.0674 (13)	0.0807 (14)	0.0840 (15)	−0.0038 (11)	0.0280 (12)	−0.0221 (12)
O5	0.0824 (14)	0.0908 (15)	0.0799 (14)	−0.0105 (12)	0.0390 (12)	−0.0273 (13)
N1	0.0765 (17)	0.0628 (16)	0.0831 (17)	0.0104 (13)	0.0320 (14)	0.0243 (13)
N2	0.0726 (16)	0.0600 (15)	0.0624 (15)	−0.0061 (13)	0.0191 (13)	0.0076 (12)
C1	0.080 (2)	0.061 (2)	0.0573 (17)	−0.0043 (17)	0.0088 (16)	0.0033 (15)
C2	0.0684 (19)	0.0625 (19)	0.0574 (17)	0.0006 (16)	0.0025 (15)	0.0046 (15)
C3	0.081 (2)	0.062 (2)	0.0541 (16)	−0.0140 (16)	0.0136 (15)	0.0024 (14)
C4	0.103 (2)	0.063 (2)	0.076 (2)	−0.0104 (18)	0.029 (2)	0.0037 (17)
C5	0.117 (3)	0.063 (2)	0.079 (2)	−0.017 (2)	0.041 (2)	0.0073 (17)
C6	0.088 (2)	0.080 (2)	0.0610 (19)	−0.0149 (18)	0.0268 (17)	−0.0039 (17)
C7	0.099 (3)	0.078 (2)	0.092 (2)	0.0024 (19)	0.042 (2)	0.0207 (19)
C8	0.094 (2)	0.083 (2)	0.079 (2)	−0.002 (2)	0.0375 (19)	0.0237 (18)
C9	0.118 (3)	0.070 (2)	0.071 (2)	−0.036 (2)	0.017 (2)	−0.0065 (18)
C10	0.077 (2)	0.073 (2)	0.0596 (18)	−0.0037 (17)	0.0122 (16)	0.0088 (16)
C11	0.086 (3)	0.081 (3)	0.126 (3)	−0.016 (2)	0.008 (2)	0.016 (2)
C12	0.072 (3)	0.136 (4)	0.199 (6)	−0.002 (3)	0.043 (3)	0.057 (4)
C13	0.103 (4)	0.137 (5)	0.164 (5)	0.042 (3)	0.049 (3)	0.035 (4)
C14	0.100 (3)	0.102 (3)	0.107 (3)	0.014 (3)	0.012 (3)	−0.005 (2)
C15	0.083 (2)	0.079 (2)	0.085 (2)	−0.013 (2)	0.0110 (19)	0.003 (2)
C16	0.0580 (16)	0.0601 (18)	0.0608 (17)	0.0017 (14)	0.0040 (14)	0.0029 (14)
C17	0.083 (2)	0.0592 (19)	0.079 (2)	0.0108 (17)	0.0112 (18)	0.0068 (16)
C18	0.073 (2)	0.065 (2)	0.087 (2)	0.0168 (16)	0.0153 (18)	−0.0068 (17)
C19	0.0598 (18)	0.063 (2)	0.073 (2)	0.0007 (15)	0.0121 (15)	−0.0117 (16)
C20	0.0617 (18)	0.0625 (19)	0.087 (2)	0.0075 (15)	0.0204 (17)	0.0120 (16)
C21	0.0599 (18)	0.0613 (19)	0.091 (2)	0.0121 (14)	0.0200 (17)	0.0127 (16)
C22	0.0583 (17)	0.0588 (17)	0.0639 (18)	0.0085 (14)	0.0151 (15)	−0.0079 (15)
C23	0.0648 (18)	0.0643 (19)	0.0612 (17)	0.0082 (15)	0.0176 (15)	−0.0084 (15)
C24	0.080 (2)	0.085 (2)	0.085 (2)	0.0062 (18)	0.0301 (19)	−0.0206 (19)
C25	0.102 (3)	0.120 (3)	0.102 (3)	0.004 (3)	0.052 (2)	−0.040 (3)
C26	0.117 (3)	0.135 (4)	0.115 (3)	−0.013 (3)	0.072 (3)	−0.025 (3)
C27	0.126 (3)	0.108 (3)	0.119 (3)	−0.032 (3)	0.072 (3)	−0.025 (3)
C28	0.102 (2)	0.078 (2)	0.087 (2)	−0.006 (2)	0.048 (2)	−0.0178 (19)

### Geometric parameters (Å, º)

**Table:**

O1—N1	1.416 (4)	C18—C19	1.368 (4)
O1—C1	1.329 (4)	C19—C20	1.375 (4)
O2—C6	1.436 (4)	C20—C21	1.388 (5)
O2—C9	1.326 (5)	C22—C23	1.482 (4)
O3—C9	1.200 (5)	C23—C24	1.383 (5)
O4—C19	1.419 (4)	C23—C28	1.386 (5)
O4—C22	1.362 (4)	C24—C25	1.391 (5)
O5—C22	1.201 (4)	C25—C26	1.369 (6)
N1—C2	1.319 (4)	C26—C27	1.368 (6)
N2—C1	1.336 (4)	C27—C28	1.379 (6)
N2—C2	1.335 (4)	C4—H4	0.9300
C1—C3	1.464 (5)	C5—H5	0.9300
C2—C16	1.471 (5)	C7—H7	0.9300
C3—C4	1.387 (5)	C8—H8	0.9300
C3—C8	1.392 (5)	C11—H11	0.9300
C4—C5	1.372 (5)	C12—H12	0.9300
C5—C6	1.373 (5)	C13—H13	0.9300
C6—C7	1.383 (5)	C14—H14	0.9300
C7—C8	1.376 (5)	C15—H15	0.9300
C9—C10	1.506 (5)	C17—H17	0.9300
C10—C11	1.387 (6)	C18—H18	0.9300
C10—C15	1.372 (5)	C20—H20	0.9300
C11—C12	1.381 (7)	C21—H21	0.9300
C12—C13	1.381 (8)	C24—H24	0.9300
C13—C14	1.342 (8)	C25—H25	0.9300
C14—C15	1.348 (6)	C26—H26	0.9300
C16—C17	1.390 (5)	C27—H27	0.9300
C16—C21	1.387 (4)	C28—H28	0.9300
C17—C18	1.372 (5)		
			
N1—O1—C1	105.6 (2)	C22—C23—C24	123.1 (3)
C6—O2—C9	118.8 (3)	C22—C23—C28	117.7 (3)
C19—O4—C22	117.0 (2)	C24—C23—C28	119.2 (3)
O1—N1—C2	103.9 (2)	C23—C24—C25	120.1 (3)
C1—N2—C2	103.1 (3)	C24—C25—C26	119.8 (4)
O1—C1—N2	112.8 (3)	C25—C26—C27	120.4 (4)
O1—C1—C3	120.7 (3)	C26—C27—C28	120.3 (4)
N2—C1—C3	126.5 (3)	C23—C28—C27	120.1 (3)
N1—C2—N2	114.6 (3)	C3—C4—H4	119.00
N1—C2—C16	120.0 (3)	C5—C4—H4	119.00
N2—C2—C16	125.4 (3)	C4—C5—H5	121.00
C1—C3—C4	122.7 (3)	C6—C5—H5	121.00
C1—C3—C8	118.6 (3)	C6—C7—H7	121.00
C4—C3—C8	118.6 (3)	C8—C7—H7	121.00
C3—C4—C5	121.5 (3)	C3—C8—H8	120.00
C4—C5—C6	118.6 (3)	C7—C8—H8	120.00
O2—C6—C5	123.6 (3)	C10—C11—H11	120.00
O2—C6—C7	114.2 (3)	C12—C11—H11	120.00
C5—C6—C7	121.8 (3)	C11—C12—H12	120.00
C6—C7—C8	118.9 (3)	C13—C12—H12	120.00
C3—C8—C7	120.6 (3)	C12—C13—H13	120.00
O2—C9—O3	125.0 (4)	C14—C13—H13	120.00
O2—C9—C10	111.0 (3)	C13—C14—H14	120.00
O3—C9—C10	124.0 (4)	C15—C14—H14	120.00
C9—C10—C11	116.5 (3)	C10—C15—H15	119.00
C9—C10—C15	124.4 (3)	C14—C15—H15	119.00
C11—C10—C15	119.1 (3)	C16—C17—H17	120.00
C10—C11—C12	119.0 (4)	C18—C17—H17	120.00
C11—C12—C13	119.6 (5)	C17—C18—H18	120.00
C12—C13—C14	120.7 (5)	C19—C18—H18	120.00
C13—C14—C15	120.2 (4)	C19—C20—H20	121.00
C10—C15—C14	121.3 (4)	C21—C20—H20	121.00
C2—C16—C17	121.2 (3)	C16—C21—H21	120.00
C2—C16—C21	119.8 (3)	C20—C21—H21	120.00
C17—C16—C21	119.0 (3)	C23—C24—H24	120.00
C16—C17—C18	120.3 (3)	C25—C24—H24	120.00
C17—C18—C19	119.8 (3)	C24—C25—H25	120.00
O4—C19—C18	118.0 (3)	C26—C25—H25	120.00
O4—C19—C20	120.2 (3)	C25—C26—H26	120.00
C18—C19—C20	121.7 (3)	C27—C26—H26	120.00
C19—C20—C21	118.4 (3)	C26—C27—H27	120.00
C16—C21—C20	120.8 (3)	C28—C27—H27	120.00
O4—C22—O5	122.5 (3)	C23—C28—H28	120.00
O4—C22—C23	112.0 (2)	C27—C28—H28	120.00
O5—C22—C23	125.5 (3)		
			
C1—O1—N1—C2	0.2 (3)	O2—C9—C10—C11	175.5 (3)
N1—O1—C1—N2	−0.8 (3)	O2—C9—C10—C15	−4.9 (5)
N1—O1—C1—C3	178.0 (3)	O3—C9—C10—C11	−5.0 (5)
C9—O2—C6—C5	59.1 (4)	O3—C9—C10—C15	174.6 (4)
C9—O2—C6—C7	−128.3 (3)	C9—C10—C11—C12	−179.5 (4)
C6—O2—C9—O3	−1.6 (5)	C15—C10—C11—C12	0.9 (6)
C6—O2—C9—C10	177.9 (3)	C9—C10—C15—C14	179.6 (3)
C22—O4—C19—C18	111.2 (3)	C11—C10—C15—C14	−0.8 (5)
C22—O4—C19—C20	−71.4 (4)	C10—C11—C12—C13	0.3 (7)
C19—O4—C22—O5	3.2 (4)	C11—C12—C13—C14	−1.6 (8)
C19—O4—C22—C23	−176.0 (2)	C12—C13—C14—C15	1.7 (7)
O1—N1—C2—C16	−178.9 (3)	C13—C14—C15—C10	−0.5 (6)
O1—N1—C2—N2	0.5 (3)	C2—C16—C17—C18	179.0 (3)
C2—N2—C1—C3	−177.6 (3)	C21—C16—C17—C18	−0.2 (5)
C1—N2—C2—C16	178.4 (3)	C2—C16—C21—C20	−179.6 (3)
C2—N2—C1—O1	1.1 (3)	C17—C16—C21—C20	−0.4 (5)
C1—N2—C2—N1	−1.0 (3)	C16—C17—C18—C19	0.0 (5)
O1—C1—C3—C8	177.1 (3)	C17—C18—C19—O4	178.2 (3)
N2—C1—C3—C4	173.5 (3)	C17—C18—C19—C20	0.8 (5)
N2—C1—C3—C8	−4.2 (5)	O4—C19—C20—C21	−178.7 (3)
O1—C1—C3—C4	−5.1 (5)	C18—C19—C20—C21	−1.5 (5)
N1—C2—C16—C17	4.3 (4)	C19—C20—C21—C16	1.2 (5)
N1—C2—C16—C21	−176.5 (3)	O4—C22—C23—C24	17.3 (4)
N2—C2—C16—C17	−175.0 (3)	O4—C22—C23—C28	−163.9 (3)
N2—C2—C16—C21	4.1 (5)	O5—C22—C23—C24	−161.9 (3)
C1—C3—C4—C5	−177.6 (3)	O5—C22—C23—C28	17.0 (5)
C8—C3—C4—C5	0.2 (5)	C22—C23—C24—C25	179.0 (3)
C1—C3—C8—C7	177.1 (3)	C28—C23—C24—C25	0.2 (5)
C4—C3—C8—C7	−0.8 (5)	C22—C23—C28—C27	−179.3 (3)
C3—C4—C5—C6	−0.2 (5)	C24—C23—C28—C27	−0.4 (5)
C4—C5—C6—O2	172.9 (3)	C23—C24—C25—C26	0.3 (6)
C4—C5—C6—C7	0.8 (5)	C24—C25—C26—C27	−0.6 (6)
O2—C6—C7—C8	−174.1 (3)	C25—C26—C27—C28	0.4 (6)
C5—C6—C7—C8	−1.3 (5)	C26—C27—C28—C23	0.1 (6)
C6—C7—C8—C3	1.3 (5)		

### Hydrogen-bond geometry (Å, º)

*Cg*3 is the centroid of the C10–C15 ring.

**Table:**

*D*—H···*A*	*D*—H	H···*A*	*D*···*A*	*D*—H···*A*
C18—H18···O5^i^	0.93	2.57	3.407 (5)	150
C25—H25···O3^ii^	0.93	2.34	3.222 (6)	158
C28—H28···*Cg*3^iii^	0.93	2.96	3.678 (5)	135

Symmetry codes: (i) *x*, *y*+1, *z*; (ii) −*x*+1, −*y*+2, −*z*+1; (iii) −*x*+1, *y*−1/2, −*z*+1/2.
